# Combined treatment with a pH-sensitive fusogenic peptide and cationic lipids achieves enhanced cytosolic delivery of exosomes

**DOI:** 10.1038/srep10112

**Published:** 2015-05-26

**Authors:** Ikuhiko Nakase, Shiroh Futaki

**Affiliations:** 1 Nanoscience and Nanotechnology Research Center, Research Organization for the 21st Century, Osaka Prefecture University, Naka-ku, Sakai, Osaka 599-8570, Japan; 2 Institute for Chemical Research, Kyoto University, Uji, Kyoto 611-0011, Japan

## Abstract

Exosomes, which are approximately 100 nm vesicles secreted by cells, have been studied with respect to cell-to-cell communication, disease diagnosis, and intracellular delivery. The cellular uptake of exosomes occurs by endocytosis; however, the cytosolic release efficiency of encapsulated molecules inside cells is low. To address this issue, here we demonstrate a simple technique for enhancing the cellular uptake and cytosolic release of exosomes by combining a pH-sensitive fusogenic peptide for the fusion of endosomal and exosomal membranes inside cells. This method stimulates the efficient cytosolic release of the exosomal contents with cationic lipids that act as a “glue” to support cellular uptake. Using this simple combined technique, the effective cellular uptake and cytosolic release of an artificially encapsulated dextran macromolecule (70 kDa) in exosomes are achieved, and a marked improvement in bioactivity is attained with the artificially encapsulated ribosome-inactivating protein saporin. Our method will contribute to many biological research fields, including the assessment of the activities of exosomal contents and the development of candidate tools enabling intracellular visualisation and cellular regulation for future therapeutic applications.

Most cells constitutively secrete exosomes, which are vesicles ~100 nm in diameter with a lipid bilayer morphology found in abundance in body fluids including blood, saliva, urine, and breast milk^1^,^2^. For example, human blood serum contains approximately 3,000,000 exosomes per microliter^2^. Because exosomes transport genetic material (e.g., microRNA) and bioactive proteins, they function in cell-to-cell communication, signal transduction for cellular regulation, and modulation of the immune response^1^,^2^,^3^. In cell-to-cell communication, exosomes also serve as vehicles for shuttling various biological molecules between cells for regulatory purposes, including membrane receptors, proteins, mRNA and microRNA^1^. Exosomes transport biofunctional materials to neighbouring cells via endocytosis, including tetraspanin membrane proteins (CD9, CD63, CD81, CD82), heat shock proteins (Hsp70, Hsp90), proteins involved in multivesicular body biogenesis (Alix, TSG101), and other bioactive proteins (GTPases, annexins, flotillin); raft-associated lipids such as cholesterol, ceramide, sphingolipids, phosphoglycerides and phospholipases are also contained in exosomes^4^,^5^. Additionally, the function of exosomal membrane proteins (e.g., CD9, CD81) as ligands for endocytosis has been reported; however, the nature of the complicated cellular uptake mechanisms are still under debate^6^,^7^,^8^,^9^. Various types of disease-related cells including tumour cells secrete exosomes that carry specific microRNAs (e.g., miR-1246 (esophageal squamous cell carcinoma), miR-1229 (colon cancer)), and detection of exosomal microRNA is considered a potential method for disease diagnosis. Therefore, diagnostic technologies based on exosomal microRNAs are being urgently developed, and these technologies are highly anticipated as promising future diagnostic methodologies, which may be applied for the early detection of hidden diseases and reducing the suffering of patients in clinical examinations^10^,^11^,^12^.

Conversely, exosomes have been recently being studied as natural nanomaterials for the delivery of bioactive genes^1^,^3^,^13^,^14^,^15^ and for therapeutic treatment including, for example, the knockdown of BACE1 as a therapeutic target in Alzheimer’s disease^16^. Exosomes have great advantages as drug delivery carriers because of very low cytotoxicity, non-immunogenicity, constitutive secretion from cells, original and artificial encapsulation of bioactive molecules (especially microRNAs), and the protein-engineering of the exosomal membrane^17^. However, because a high amount of exosomes in bodily fluids induces competition for their cellular uptake by endocytosis^8^,^18^, the cellular uptake efficiency of exosomal vehicles for intracellular delivery is considered insufficient for therapeutic treatment. New technology to enhance the cytosolic release of exosome contents is also strongly needed to achieve effective biological and therapeutic activities of molecules contained in exosomes inside targeted cells. When exosomes are taken up by cells and trafficked by endosomal pathways, the exosome contents suffer from molecular digestion during the process of endosomal maturation, and the biological activities of the exosome contents might be reduced before their release from exosomes and endosomes inside cells. Recently, cellular targeting of exosomes by fusion of cell receptor recognition proteins such as rabies viral glycoprotein (RVG)^16^ and integrin-specific iRGD peptide for αv integrin targeting^19^ with exosomal membrane proteins was reported; however, new techniques for enhanced cellular uptake and the cytosolic release of exosomal contents need to be developed to achieve sophisticated delivery vehicles.

In this report, we propose a simple and effective technique for enhancing the cytosolic release of exosomal contents using commercially available cationic lipids and a pH-sensitive fusogenic peptide (Fig. 1). We previously reported that a combination of cationic lipids and a pH-sensitive fusogenic peptide, GALA, significantly enhanced endosomal release of proteins that were conjugated with the GALA peptide^20^,^21^ into the cytosol, and we have now applied this methodology for enhanced cytosolic delivery of exosomal contents. The addition of a commercially available cationic lipid formulation, Lipofectamine LTX, highly enhances the cellular uptake of CD63-green fluorescent protein (GFP)-tagged exosomes without any cytotoxicity by combining the cationic lipid treatment with exosomes that have a negatively-charged exosomal membrane. We also achieved the efficient cytosolic release of exosomal contents in an endocytotic pathway using the GALA peptide by the combination of exosomes and cationic lipids that work as “glue” to accumulate targeted cellular membranes and enhance cellular uptake and cytosolic release (Fig. 1). The cellular uptake of artificially encapsulated dextran (70 kDa) in exosomes was significantly enhanced by the combined treatment, leading to the efficient cytosolic release of encapsulated dextran from exosomes inside cells. In addition, intracellular delivery of a ribosome-inactivating protein, saporin, using exosomes was also achieved using cationic lipids and GALA peptides, leading to the efficient induction of cytotoxicity in targeted cells.

## Results

### Effect of cationic lipids on cellular uptake of exosomes

**Figure 1:**
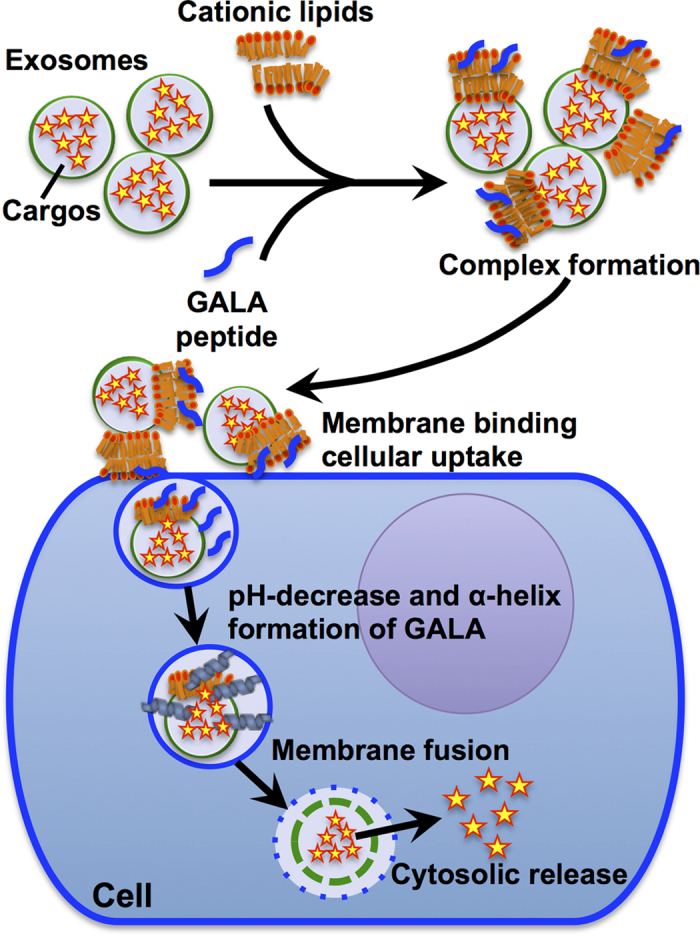
Schematic representation of the cytosolic delivery of exosomal cargos with a combination of cationic lipids and the pH-sensitive fusogenic GALA peptide. Cationic lipids and the GALA peptide enhance cell membrane binding and the cellular uptake of exosomes. After cellular uptake by endocytosis, a pH decrease in the endosomes increases the helical content of the GALA peptide, leading to fusion of endosomal and exosomal membranes in cells and the cytosolic release of exosomal cargos.

As mentioned in the Introduction, exosomes participate in cell-to-cell communication by secretion and the cellular uptake of exosomes loaded with regulatory molecules (e.g., microRNAs). However, negatively charged exosomal membranes^22^,^23^,^24^ repel negatively charged cellular membranes. Because the high abundance of exosomes in serum also leads to competition for their cellular uptake, the internalisation efficiency of exosomes into cells is low and slow. Therefore, further development of strategies for the enhanced cellular uptake of exosomes is urgently required for the delivery of therapeutic molecules. We examined the cellular uptake of exosomes using a commercially available transfection reagent, Lipofectamine LTX, which contains cationic lipids that can act as a “glue” for pasting exosomes to targeted cells (Fig. 1). We studied exosomes that have a negatively charged membrane. Tetraspanin CD63 protein is a marker of exosomal membrane protein, and green fluorescent protein (GFP)-fused CD63 stably-expressing HeLa cells (CD63-GFP-HeLa) (Supplementary Fig. 1a) were prepared as described in the Methods section. Secreted CD63-GFP-exosomes were isolated from the cell culture medium using the Total Exosome Isolation kit, and expression of the exosomal marker proteins CD63 fused with GFP (Supplementary Fig. 1b and c) and CD9 (Supplementary Fig. 1d) was detected by western blot analysis. Exosomal morphology was also observed by transmission electron microscopy (TEM) (Supplementary Fig. 1e).

**Figure 2:**
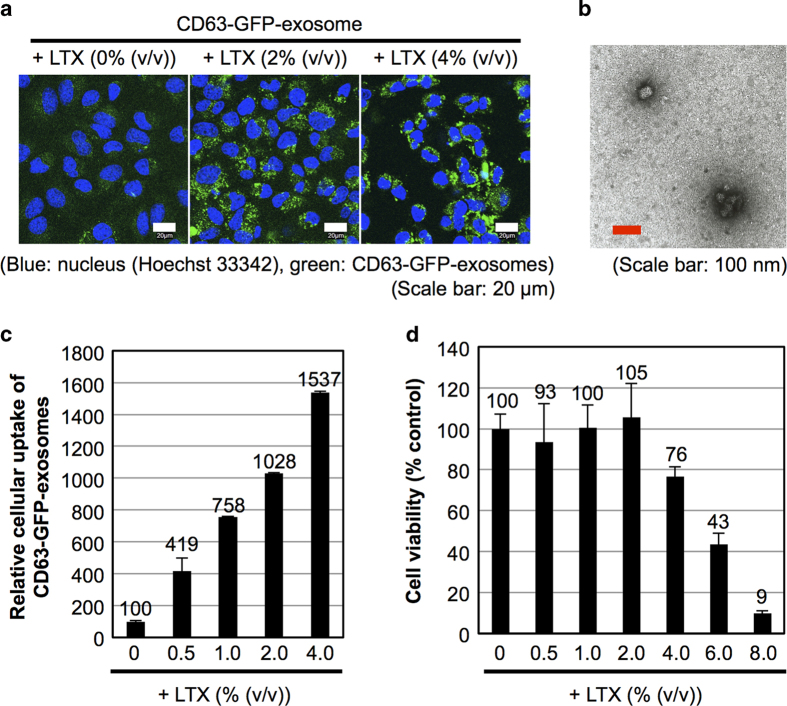
Increased cellular uptake of exosomes by treatment with cationic lipids. (**a**) Confocal microscopic observation of HeLa cells treated with CD63-GFP-exosomes (20 μg/ml) in the presence or absence of Lipofectamine LTX (2 or 4% (v/v)) for 24 h at 37 °C (blue: Hoechst33342, green: CD63-GFP-exosomes). Scale bar: 20 μm. (**b**) TEM observation of CD63-GFP-exosomes (20 μg/ml) mixed with Lipofectamine LTX (2% (v/v)). Scale bar: 100 nm. (**c**) Relative cellular uptake of CD63-GFP-exosomes (20 μg/ml) in the presence or absence of Lipofectamine LTX (0.5–4% (v/v)) analysed using a flow cytometer under the same experimental conditions as (**a**). (**d**) Cytotoxicity in the combined treatment of exosomes (20 μg/ml) and Lipofectamine LTX (0.5–8% (v/v)) for 24 h at 37 °C of HeLa cells analysed by the WST-1 assay. The data are the averages (±SD) of three (**c**) and four (**d**) experiments.

HeLa cells were treated with the CD63-GFP-exosomes (20 μg/ml of proteins) in α-minimal essential medium (MEM) containing 10% foetal bovine serum (FBS) for 24 h at 37 °C, but the fluorescent signals of the exosomes inside the cells were difficult to observe using a confocal microscope because of the very low cellular uptake efficacy under these conditions (Fig. 2a and Supplementary Fig. 2a).

We next examined the effect of cationic lipids on the cellular uptake of the exosomes. The CD63-GFP-exosomes were mixed with a commercially available cationic lipid formulation, Lipofectamine LTX, which has been widely used for gene transfection^25^ as described in the Methods section. The zeta-potential of the exosome/LTX complex was shown to be −1.15 mV as analysed using a zeta-potential analyser. The average diameter of the exosomes was 78 nm as analysed by TEM (Fig. 2b). Conversely, the average diameter of the original isolated exosomes without the addition of Lipofectamine LTX was 57 nm, as analysed by TEM (Supplementary Fig. 1e). The zeta-potential of isolated exosomes was shown to be −11.58 mV as analysed using a zeta-potential analyser, which was similar to previously reported results^22^,^23^,^24^, suggesting a reduction of the negative charge of the exosomal membrane by the addition of Lipofectamine LTX.

The cellular uptake efficiency of the CD63-GFP-exosomes in combination with Lipofectamine LTX was analysed using a confocal microscope (Fig. 2a and Supplementary Fig. 2a) and a flow cytometer (Fig. 2c). HeLa cells were treated with CD63-GFP-exosomes (20 μg/ml of proteins) and Lipofectamine LTX (0–4.0% (v/v)) in α-MEM containing 10% FBS for 24 h at 37 °C, prior to confocal microscopic analysis. The addition of Lipofectamine LTX was shown to significantly affect the cellular uptake of CD63-GFP-exosomes, and for example, treatment with 4.0% (v/v) Lipofectamine LTX increased the internalisation of the exosomes into HeLa cells by approximately 15-fold (Fig. 2c), suggesting that the addition of Lipofectamine LTX effectively enhances the cellular uptake efficiency of exosomes. Colocalization of endosome marker DiD and internalized CD63-GFP-exosomes was observed by the combined treatment with Lipofectamine LTX (Supplementary Fig. 2b). However, a WST-1 assay for the detection of cell viability showed that a high concentration of Lipofectamine LTX induced cytotoxicity (e.g., treatment of 8.0% Lipofectamine LTX resulted in ~90% cell death) (Fig. 2d), suggesting that it was necessary to optimise the cationic lipid content for the cellular uptake efficiency of exosomes versus cytotoxicity. The effect of cationic lipids on the cellular uptake of exosomes was also examined in CHO-K1 cells (Supplementary Fig. 3). Under the same experimental conditions as described for Fig. 2a,c, the cellular uptake efficiency of CD63-GFP-exosomes was significantly increased by a combination including Lipofectamine LTX. For example, Lipofectamine LTX (4.0% (v/v)) increased cellular uptake efficiency of exosomes by approximately 175-fold by CHO-K1 cells (Supplementary Fig. 3a and b). Conversely, a high concentration of Lipofectamine LTX also enhanced cytotoxicity (Supplementary Fig. 3c). A combination treatment with Lipofectamine LTX for CD63-GFP-exosomes that were isolated by ultracentrifugation as described in the Methods section was also tested, and similar results showing enhanced cellular uptake efficiency of the exosomes were observed (e.g., approximately 11-fold enhanced cellular uptake by the combination treatment of 2.0% (v/v) Lipofectamine LTX under the same experimental conditions as described for Fig. 2c).

Cellular uptake of CD63-GFP-exosomes at lower concentrations (1–10 μg/ml of protein) was also significantly enhanced by addition of Lipofectamine LTX. For example, ~6-fold enhancement in cellular uptake of CD63-GFP-exosomes (5 μg/ml) was attained by the combination treatment with 2.0% (v/v) Lipofectamine LTX (Supplementary Fig. 4a). However, as in the case of Fig. 2d, the addition of excess Lipofectamine LTX yielded considerable cell death (Supplementary Fig. 4b).

### Cellular uptake of a pH-sensitive fusogenic peptide, GALA, treated with exosomes and cationic lipids

Endocytosis has been shown to be a major pathway for the cellular uptake of exosomes^6^,^7^,^8^,^9^, and barriers imposed by endosomal and exosomal membranes interfere with the cytosolic release of exosomal contents. Therefore, the cytosolic release efficiency of the exosomal contents (e.g., genes and proteins) greatly influences their bioactivities in targeted cells. We previously reported that a pH-sensitive fusogenic peptide, GALA, can enhance the disruption of the endosomal membrane, leading to the efficient cytosolic release of proteins that were conjugated with the GALA peptide inside of the cells^20^,^21^. A 30-residue amphipathic peptide with a repeating sequence of glutamic acid-alanine-leucine-alanine (the amino acid sequence of GALA is WEAALAEALAEALAEHLAEALAEALEALAA) was designed to mimic a viral fusion protein sequence that mediates the escape of viral genes from acidic endosomes to the cytosol^26^,^27^. The GALA peptide converts the structure from random to helical when the pH is reduced from 7.5 to 5.0, leading to membrane fusion^26^,^27^. Cationic lipids were also employed to cause the accumulation of the GALA peptide, which carries a negative charge from glutamic acids (7 residues), on the cell surface and accelerate cellular uptake^20^,^21^. In this research, we investigated an application of the GALA peptide in a combined treatment of exosomes with cationic lipids for the fusion of exosomal and endosomal membranes inside cells and the enhanced cytosolic release of exosomal contents (Fig. 1).

**Figure 3:**
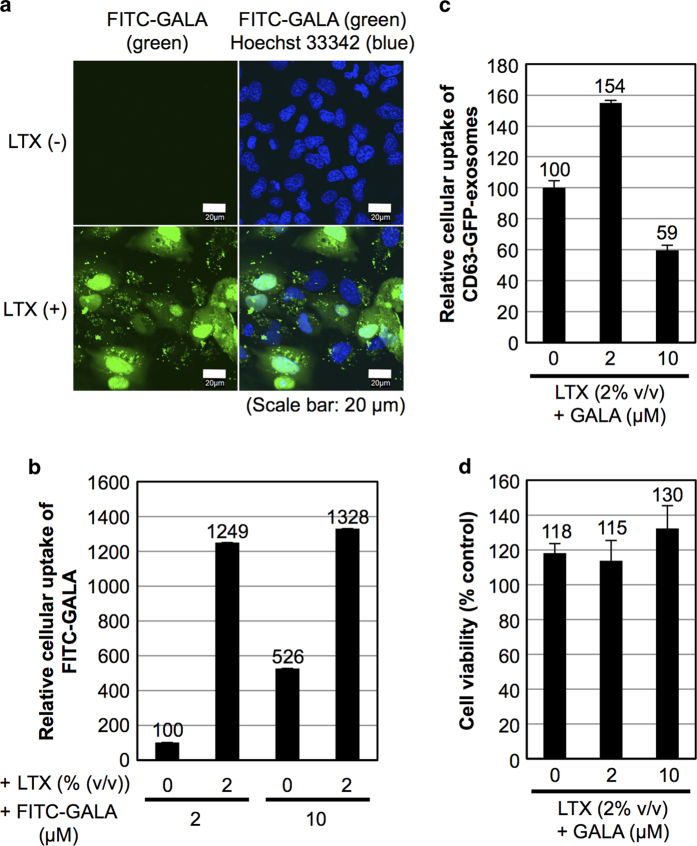
Cellular uptake and cytosolic release of FITC-GALA in combination with cationic lipids. (**a**) Confocal microscopic observation of HeLa cells treated in combination with FITC-GALA (2 μM) and exosomes (without expression of CD63-GFP, 20 μg/ml) in the presence or absence of Lipofectamine LTX (2% (v/v)) for 6 h at 37 °C (blue: Hoechst33342, green: FITC-GALA). Scale bar: 20 μm. (**b**) Relative cellular uptake of FITC-GALA (2 or 10 μM) with exosomes (without expression of CD63-GFP, 20 μg/ml) in the presence or absence of Lipofectamine LTX (2% (v/v)) analysed using a flow cytometer. (**c**) Relative cellular uptake of CD63-GFP-exosomes (20 μg/ml) with Lipofectamine LTX (2% (v/v)) in the presence or absence of GALA peptide (without fluorescent label, 2 or 10 μM) analysed using a flow cytometer. Experiments corresponding to (**b**) and (**c**) were conducted under the same experimental conditions as (**a**). (**d**) Cytotoxicity of the combination treatment of exosomes (20 μg/ml) and Lipofectamine LTX (2% (v/v)) in the presence or absence of GALA (2 or 10 μM) for 24 h at 37 °C of HeLa cells analysed by a WST-1 assay. The data are the averages (±SD) of three (**b**, **c**) and four (**d**) experiments.

Figure 3a and b show the cellular uptake of FITC-labelled GALA (FITC-GALA) peptides. HeLa cells were treated with exosomes (without expression of CD63-GFP, 20 μg/ml) and FITC-GALA (2 μM) in the presence or absence of Lipofectamine LTX (2% (v/v)) in α-MEM containing 10% FBS for 6 h at 37 °C, prior to analysis by confocal microscopy (Fig. 3a) and flow cytometry (Fig. 3b). The addition of Lipofectamine LTX significantly enhanced the cellular uptake efficiency of the FITC-GALA peptide, and diffuse fluorescent signals from GALA in the cytosol and the nucleus were observed (Fig. 3a). Conversely, very little cellular uptake of FITC-GALA was observed in the absence of Lipofectamine LTX (Fig. 3a). Analysis using a flow cytometer showed the increased cellular uptake of FITC-GALA (2 μM) in a combined treatment with Lipofectamine LTX (2% (v/v)) (~12-fold), and a high concentration of FITC-GALA (10 μM) produced a cellular uptake efficiency similar to that of 2 μM FITC-GALA in the presence of Lipofectamine LTX (2% (v/v)) (Fig. 3b). The advantage of the combined treatment of GALA and Lipofectamine LTX to improve the cytosolic release of exosomal contents was also confirmed in Fig. 3c, showing the relative cellular uptake of CD63-GFP-exosomes with Lipofectamine LTX (2% (v/v)) in the presence or absence of GALA peptide (without fluorescent label, 2 or 10 μM) under the same experimental conditions as described in Fig. 3a, analysed using a flow cytometer. The addition of the GALA peptide (2 μM) resulted in enhanced cellular uptake of CD63-GFP-exosomes (approximately 1.5-fold). Alternatively, the addition of 10 μM GALA peptide decreased the cellular uptake of CD63-GFP-exosomes, suggesting that a high concentration of the GALA peptide decreases the internalisation efficiency of exosomes because the excess negative charge of the peptide might affect cellular uptake (Fig. 3c). Cytotoxicity was also examined as described in Methods section, and the addition of the GALA peptide did not reduce cell viability as analysed by the WST-1 assay (Fig. 3d), suggesting that the induction of endosomal and exosomal fusion by the pH-sensitive fusogenic peptide has advantages related to cytotoxicity.

The effect of FITC-GALA (0.05, 0.5 or 2.5 μM) was also analysed when the concentration of exosomes (without expression of CD63-GFP) was reduced to 5 μg/ml in the combined treatment with Lipofectamine LTX (0.5% (v/v)) (Supplementary Fig. 5a). FITC-GALA (0.5 μM) showed its effective diffusion into cytosol and nucleus (Supplementary Fig. 5a). Conversely, no cytosolic diffusion was observed by the treatment with 0.05 or 2.5 μM FITC-GALA (Supplementary Fig. 5a). These results suggested that there should be an optimal concentration of GALA peptide for each Lipofectamine LTX concentration to achieve effective cytosolic release as previously reported by our group^20^,^21^. In contrast to this result of significant cytosolic FITC-GALA signal, CD63-GFP-exosomes instead showed punctuate, endosome-like structures, suggesting the retention of CD63-GFP-exosomes in endosomes or the fusion of CD63-GFP-exosomal membranes into endosomes (Supplementary Fig. 5b). When endosomal pH-reduction was prevented by treatment with NH_4_Cl (50 mM)^20^, cytosolic diffusion of FITC-GALA peptide was significantly suppressed (Supplementary Fig. 5c), suggesting that endosomal pH-reduction is essential for GALA-mediated membrane fusion between exosomes and endosomes to attain cytosolic release of exosomal contents.

### Cellular uptake of dextran-encapsulated exosomes and enhanced cytosolic release by cationic lipids and GALA peptide

Next, we examined the cytosolic delivery of cargo-encapsulated exosomes using the combined treatment consisting of the cationic lipids and the GALA peptide. Dextran (70 kDa) was adopted as a model macromolecular cargo in this experiment. It is difficult for dextran to penetrate through the cell membrane by itself. We used Texas red-labelled dextran (TR-dex) to visualise the intracellular localisation of encapsulated dextran in exosomes delivered using the combined treatment with the cationic lipids and the GALA peptide. We used an optimized electroporation condition to encapsulate TR-dex into exosomes as described in the Methods section. After the cargo-loading via electroporation, removal of unencapsulated TR-dex was conducted by repeatedly washing with PBS and filtration with centrifugal filters.

**Figure 4:**
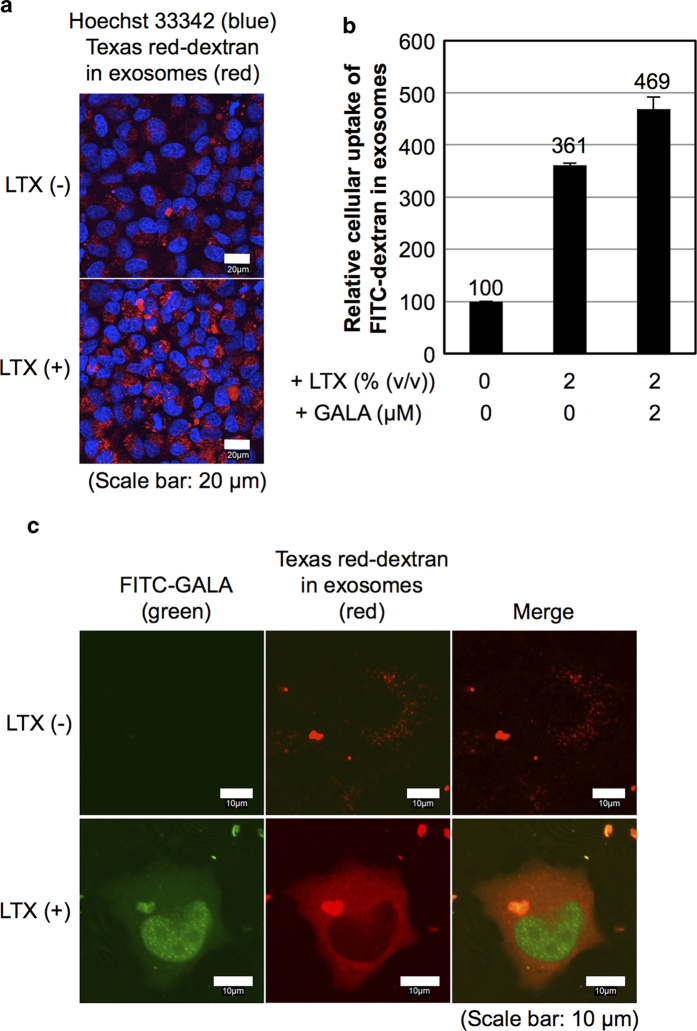
Enhanced cytosolic release of encapsulated cargos in exosomes upon a combination treatment with cationic lipids and GALA peptide. (**a**) Confocal microscopic observation of HeLa cells treated with TR-dex encapsulated exosomes (20 μg/ml) in the presence or absence of Lipofectamine LTX (2% (v/v)) for 6 h at 37 °C (blue: Hoechst33342, red: TR-dex). Scale bar: 20 μm. (**b**) Relative cellular uptake of FITC-dex encapsulated exosomes (20 μg/ml) in the presence or absence of GALA peptide (2 μM) and Lipofectamine LTX (2% (v/v)) analysed using a flow cytometer under the same experimental conditions as (**a**). The data represent the averages (±SD) of three experiments. (**c**) Confocal microscopic observation of HeLa cells treated with a combination of TR-dex encapsulated exosomes (20 μg/ml) and FITC-GALA (2 μM) in the presence or absence of Lipofectamine LTX (2% (v/v)) for 6 h at 37 °C (green: FITC-GALA, red: TR-dex). Scale bar: 10 μm.

HeLa cells were treated with TR-dex-encapsulated exosomes (exosome: 20 μg/ml, encapsulated TR-dex: 150 ng/ml) in combination with Lipofectamine LTX (2% (v/v)) in α-MEM containing 10% FBS for 24 h at 37 °C, prior to analysis using a confocal microscope. Significantly increased cellular uptake of TR-dex-encapsulated exosomes was observed upon the addition of Lipofectamine LTX (Fig. 4a). Analysis using a flow cytometer showed increased cellular uptake of FITC-dextran (FITC-dex)-encapsulated exosomes (exosome: 20 μg/ml, encapsulated FITC-dex: 100 ng/ml) (approximately 3.6-fold) in combination with Lipofectamine LTX (2% (v/v)) (Fig. 4b). Furthermore, HeLa cells were treated with TR-dex encapsulated exosomes (exosome: 20 μg/ml, encapsulated TR-dextran: 150 ng/ml) in combination with Lipofectamine LTX (2% (v/v)) and FITC-GALA (2 μM) in α-MEM containing 10% FBS for 6 h at 37 °C, prior to analysis using confocal microscope. Diffuse fluorescent signals of TR-dextran were observed in the cytosol in approximately 60% of the cells, which were accompanied by the cytosolic release of FITC-GALA (Fig. 4c). Conversely, in the absence of Lipofectamine LTX, the cellular uptake of FITC-GALA was difficult to observe, and only endosome-like punctuate signals of TR-dex were noted in the cells (Fig. 4c). In addition, significant cytosolic release of TR-dex was observed even when a lower concentration of TR-dex-encapsulated exosomes (exosome: 5 μg/ml, encapsulated TR-dex: 38 ng/ml) was employed in the presence of Lipofectamine LTX (0.5% (v/v)) and FITC-GALA (0.5 μM) (Supplementary Fig. 5d). Prevention of endosomal pH-reduction by NH_4_Cl (50 mM) decreased cytosolic release efficacy of TR-dex (Supplementary Fig. 5d), suggesting that pH-reduction in endosomes is essential for membrane fusion of exosomes with endosomes in the presence of the GALA peptide and the eventual cytosolic release of FITC-GALA and exosomal contents. We also examined the cytosolic release of dextran-encapsulated exosomes in CHO-K1 cells (Supplementary Fig. 6). In the case of the CHO-K1 cells, the addition of Lipofectamine LTX (2% (v/v)) and FITC-GALA (2 μM) enhanced the cytosolic release of TR-dextran similar to that in HeLa cells (Supplementary Fig. 6). Conversely, an increase in FITC-GALA concentration (10 μM) diminished cytosolic release of TR-dex (Supplementary Fig. 6). In addition, when HeLa cells were treated with TR-dex (150 ng/ml) without encapsulation in exosomes under the same experimental conditions as in Fig. 4c, analysis using a confocal microscope showed that it was difficult to observe the cellular localisation of TR-dex. This difficulty occurred due to the low level of fluorescence intensity of the TR-dex signals in the cells, suggesting that encapsulation in exosomes significantly enhances the cellular uptake of dextran.

### Intracellular delivery of saporin-encapsulated exosomes with combined treatment of cationic lipids and GALA peptide

**Figure 5:**
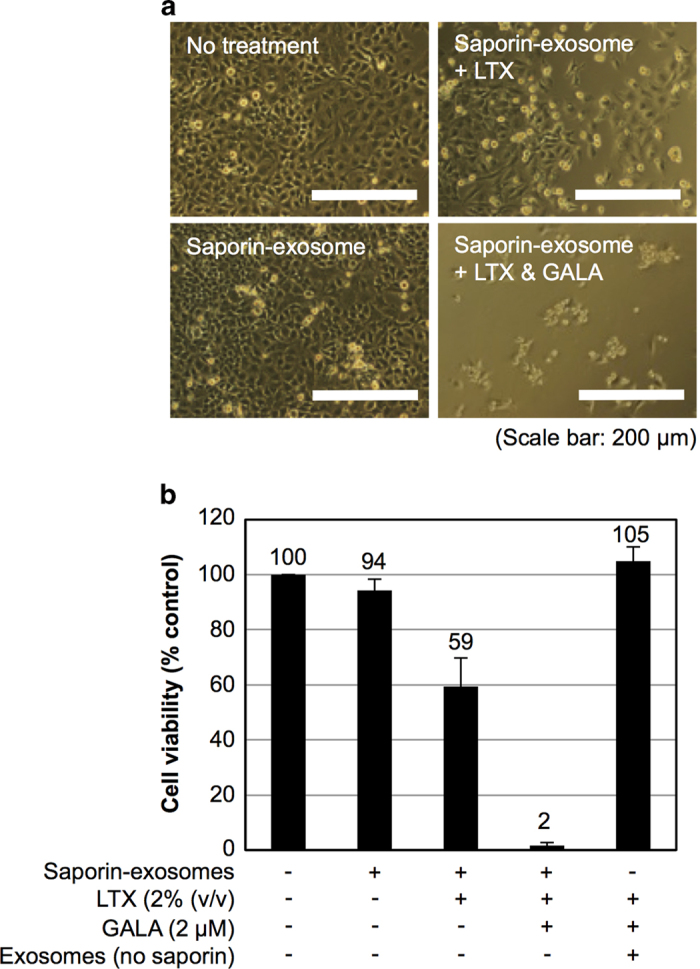
Combined treatment of saporin-encapsulated exosomes with cationic lipids and GALA peptide effectively induces cytotoxicity. (**a**) Microscopic observation of HeLa cells treated with saporin-encapsulated exosomes (20 μg/ml) in the presence or absence of Lipofectamine LTX (2% (v/v)) and GALA peptide (2 μM) for 24 hr at 37 °C. Scale bar: 200 μm. (**b**) Cytotoxicity of HeLa cells treated with saporin-encapsulated exosomes (20 μg/ml) or exosomes (20 μg/ml) without saporin in the combination treatment of Lipofectamine LTX and GALA peptide under the same experimental conditions as (**a**), analysed by the WST-1 assay. The data are the averages (±SD) of three experiments.

The delivery of saporin (approximately 30 kDa) into cells using exosomes and their biological activities were also analysed in Fig. 5 to assess the effects of the combination treatment on cytosolic delivery systems. Saporin is a ribosome-inactivating protein that inhibits protein synthesis and the growth of targeted cells^28^. In this study, saporin-encapsulated exosomes were prepared, and the induction of cell death by the combined treatment with the cationic lipids and the GALA peptide was detected. Saporin was encapsulated in exosomes by electroporation under the same experimental conditions as dextran in Fig. 4, and the removal of unencapsulated saporin was conducted using repeated centrifugal filtration as described in the Methods section. HeLa cells were treated with saporin-encapsulated exosomes (20 μg/ml) with the combined treatment with Lipofectamine LTX (2% (v/v)) and GALA peptide (2 μM). After the treatment for 24 hr, the morphology of the HeLa cells treated with the test samples was observed using a microscope, and cell viability was also assessed by the WST-1 assay (Fig. 5). Figure 5a shows a microscopic observation of the treated cells, and morphological changes in the HeLa cells were significantly induced by the combined treatment of saporin-encapsulated exosomes with Lipofectamine LTX and GALA peptide. Conversely, saporin-encapsulated exosomes without GALA peptide also induced morphological changes in the cells; however, their effects were observed to be weaker in comparison to the combined treatment with Lipofectamine LTX and GALA peptide (Fig. 5a). The effects of saporin-encapsulated exosome (20 μg/ml) treatment alone on cell morphology were difficult to observe (Fig. 5a). In the WST-1 assay for cytotoxicity, the combined treatment of saporin-encapsulated exosomes with Lipofectamine LTX and GALA peptide resulted in the highest induction of cell death (approximately 98%) in comparison to other treatment methods in this experiment (Fig. 5b). Thus, our simple combined method successfully demonstrated the effective intracellular delivery of saporin and consequent biological activity in cells by artificial encapsulation of the protein into exosomes.

## Discussion

Endocytosis has been shown to be a major pathway for the cellular uptake of exosomes for cell-to-cell communication and their use in drug delivery^6^,^7^,^8^,^9^. Live-cell microscopic imaging indicated that exosomes were taken up by cells via an endocytosis pathway by becoming trapped in vesicles and transported to lysosomes^8^. The binding of exosomes to the surface of a recipient cell involves the interaction of the exosomal membrane molecules and cellular receptors, including intracellular adhesion molecule 1 (ICAM1), lymphocyte function-associated antigen 1 (LFA1), phosphatidylserine binding to T cell immunoglobulin domain and mucin domain protein 1 (TIM1) or TIM4^7^. Svensson and coworkers also reported that the cellular uptake of exosomes was dependent on extracellular signal-regulated kinase-1/2 (ERK1/2) and heat shock protein 27 (HSP27) signalling, and ERK1/2 phosphorylation was negatively influenced by caveolin-1 during internalisation of exosomes^9^. However, complicated cellular uptake mechanisms are still under debate. Both endosomal and exosomal membranes consist of a lipid bilayer, and the exosomal contents need to penetrate the lipid layers, which are barriers against their cytosolic release and their achievement of intracellular bioactivities. Because a high number of exosomes exists in human body fluids (e.g., blood, saliva, urine), there is also intense competition for the cellular uptake of exosomes.

Parolini reported that the membrane fusion of exosomes was influenced by low pH, and acidity in melanoma cells was involved in exosomal release and cellular uptake by fusion with cell membranes^29^. The report also showed that biophysical analysis of membrane parameters such as fluidity and lipid composition indicated high rigidity and that sphingomyelin/ganglioside GM3 (*N*-acetylneuraminylgalactosylglucosylceramide) in exosomes is released at low pH^29^. However, our research showed that the release of TR-dex from the exosomes to the cytosol was difficult as shown in Fig. 4c. Differences in membrane composition in each exosome may affect the cellular uptake and membrane fusion due to pH-sensitivity; however, further studies on cellular uptake and membrane fusion mechanisms in exosomes are necessary. Considering the research results, the development of experimental techniques for the effective release of exosomal contents (e.g., genes and proteins) through endosomal and exosomal membranes should be required for bioactivity to occur inside cells before the degradation of the exosomal contents in the endosomal maturation pathway.

Recently, exosomal contents secreted from disease-related cells have been studied to understand their biological functions in cell-to-cell communication and disease progress^1^,^2^,^3^. However, experimental trials using exosomes usually take a long time to detect cellular responses because of the competitive cellular uptake of exosomes and the low cytosolic release of exosomal contents. Therefore, regarding the research objectives of drug delivery and the biological elucidation of exosomal functions, development of cytosolic release techniques is essential for understanding their biology. In this study, we adopted Lipofectamine LTX as a model cationic lipid for an *in vitro* assay for the enhancement of exosomal cellular uptake. Other types of functional cationic materials, including biocompatible and biodegradable polymers, might be applicable to our method as well, possibly opening further avenues for *in vivo* applications.

Cytosolic release from endosomes by the disruption of the endosomal membrane is considered reasonable to attain lower cytotoxicity in comparison to the disruption of plasma membranes. Because almost all enzymes for molecular digestion (e.g., of genes, proteins, sugars) show biological activity at low pH (i.e., lysosomes), enzymes released into the cytosol show little activity^30^. Therefore, the cytosolic release of enzymes induced by endosomal disruption causes little damage in the cytosol (pH of approximately 7.2), resulting in little cytotoxicity. To enhance endosomal disruption, a pH-sensitive fusogenic peptide, GALA, was adopted in this research to accelerate the cytosolic release of exosomal and endosomal contents by the fusion of these membranes.

GALA peptides have been applied to endosomal release, especially in gene transfection^31^,^32^,^33^,^34^,^35^. Our research group also successfully achieved the cytosolic delivery of peptides and proteins using the GALA peptide in a combined treatment with cationic lipids^20^,^21^. The GALA peptide was designed to mimic viral fusion protein sequences that interact with cell membranes to mediate the escape of the viral genes from acidic endosomes. Glutamic acid residues in the peptide provide a pH-dependent negatively charged side-chain, and the repeat sequence Glu-Ala-Leu-Ala has a hydrophobic face to interact with membranes when the peptide forms an α-helix. The GALA peptide converts to an amphipathic α-helix when the pH is reduced from 7.5 to 5.0, and the peptide shows a high affinity to neutral and negatively charged membranes. GALA induces fusion between small unilamellar vesicles composed of unsaturated phospholipids, and the peptide was shown to possibly form a transmembrane peptide-based pore composed of approximately ten α-helical monomers^36^,^37^,^38^. GALA peptide has been used as an additive to improve transfection efficiency and to enhance the escape of nucleic acids from endosomes.

We adopted the GALA peptide to enhance the cytosolic release of exosomal contents in a combined treatment with cationic lipids. Effective cellular uptake of FITC-GALA was observed in this treatment method, and the cytosolic release of the peptide was observed even in the presence of exosomes (Fig. 3a), suggesting the high possibility that the GALA peptide could be effectively used for membrane fusion in an exosomal delivery system. However, the cellular uptake efficiency for exosomes was decreased by a high concentration of the GALA peptide (Fig. 3c). This result is similar to our previous reports on protein delivery because charges on the peptide and cationic lipids affect their cellular uptake and membrane fusion^20^,^21^. In Supplementary Figs. 4a and 5a, concentrations of exosomes, Lipofectamine LTX, and FITC-GALA affect their cellular uptake efficiency and cytosolic release. Therefore, an optimal combined treatment using cationic lipids and the GALA peptide is important to attain an effective balance of internalisation and cytosolic release. Additionally, excess volumes of cationic lipids induce substantial cytotoxicity (Fig. 2d and Supplementary Fig. 4b), and an optimal balance of cellular uptake efficiency and cytotoxicity in the combined treatment of exosomes should be considered.

In Fig. 4, we demonstrated the effective intracellular delivery of fluorescently labelled dextran by encapsulation into exosomes and cytosolic release of the exosomal contents. As described in the Methods section, the poring pulse electroporation method affects encapsulation efficacy. Therefore, optimization of the method is needed to attain efficient encapsulation of objective molecules in exosomes. Conversely, the same concentration of dextran unencapsulated in exosomes showed a much lower cellular uptake (approximately 130-fold) than that of encapsulated dextran, suggesting that encapsulation of the dextran enhanced cellular uptake. Encapsulation of the dextran in exosomes may make changes of the original cellular uptake pathways of dextran, and warping with exosomal membranes takes advantage of compulsory internalisation by cells through exosomal cellular uptake pathways.

In addition, we also successfully achieved enhancement of the biological activity of encapsulated saporin by the combined treatment with cationic lipids and the GALA peptide (Fig. 5). Both the cationic lipids and the GALA peptide are essential for enhanced saporin activity in the induction of effective cell death resulting from treatment with saporin-encapsulated exosomes. However, in an assessment using TR-dex-encapsulated exosomes with our current method, the cytosolic release of TR-dextran from exosomes that were treated with the cationic lipids and the GALA peptide was observed in only approximately 60% of the cells. Improvement of the efficiency of the cytosolic release of the exosomal contents without cytotoxicity is a future research objective.

In conclusion, we used a simple methodology for the enhanced cellular uptake of exosomes and the efficient cytosolic release of encapsulated cargo by exosomes via the formation of a complex with cationic lipids and a pH-sensitive fusogenic peptide. Although further studies are needed for a more advanced and sophisticated development of cellular uptake and cytosolic release technology in exosomes, our method using the combined treatment will greatly contribute to many biological, pharmaceutical, and medical research fields, including the assessment of unknown activities of exosomal contents (e.g., bioactive proteins and genes) derived from disease-related cells. It will also contribute to the development of tools enabling intracellular visualisation and cellular regulation for future therapeutic applications.

## Methods

### Peptide synthesis and fluorescence labelling

The pH-sensitive fusogenic peptide, GALA (amino acid sequence: WEAALAEALAEALAEHLAEALAEALEALAA-amide), was chemically synthesised by 9-fluorenylmethyloxycarbonyl solid-phase peptide synthesis on Rink amide resin as described previously^20^,^21^. Amino acid derivatives and the Rink amide resin were purchased from the Peptide Institute (Osaka, Japan) and Shimadzu Biotech (Kyoto, Japan). Deprotection of the peptide and cleavage from the resin were conducted via treatment with a trifluoroacetic acid/ethanedithiol mixture (95:5) at room temperature for 3 h, followed by reverse-phase high-performance liquid chromatographic (HPLC) purification. For preparation of the fluorescently-labelled peptides, a 4-aminobutanoic acid residue was introduced at the N-terminus of the peptide, and fluorescence-labelling was conducted by treatment with 3 equivalents of fluorescein isothiocyanate (FITC) isomer I (Sigma-Aldrich, St. Louis, MO, USA) in *N,N*-diisopropylethylamine (3 equivalents) (Sigma-Aldrich) in N,N-dimethylformamide for 6 h at room temperature on the resin. The structures of the synthesised peptides were confirmed by matrix-assisted laser desorption ionisation time-of-flight mass spectrometry (MALDI-TOFMS) (Microflex, Bruker, Billerica, MA, USA).

### Cell culture

HeLa (human cervical cancer-derived) cells were purchased from the Riken BRC Cell Bank (Ibaraki, Japan). Chinese hamster ovary (CHO)-K1 cells were purchased from the American Type Culture Collection (Manassas, VA, USA). Each cell was cultured in α-MEM (Gibco, Life Technologies Corporation, Grand Island, NY, USA) and F-12 nutrient mixture (Ham’s F-12) (CHO-K1) (Gibco, Life Technologies Corporation) containing 10% heat-inactivated FBS (Gibco, Life Technologies Corporation). Cells were grown on 100 mm dishes and incubated at 37 °C under 5% CO_2_.

### Preparation of HeLa cells stably expressing green fluorescent protein (GFP)-fused CD63

Tetraspanin CD63 is a membrane marker protein of exosomes, and we prepared HeLa cells stably expressing GFP-fused CD63 to secrete CD63-GFP-containing exosomes (CD63-GFP-exosomes). The cells (1 × 10^5^ cells) were plated on a 24 well microplate (Iwaki, Tokyo, Japan) and incubated for 1 day. They were transfected with CD63-GFP plasmid (pCT-CD63-GFP, pCMV, Cyto-Tracer, System Biosciences, Mountain View, CA) (800 ng) complexed with Lipofectamine LTX reagent (2 μl) with PLUS reagent (1 μl) (Invitrogen, Life Technologies Corporation) in α-MEM containing 10% FBS (200 μl). The cells were also treated with puromycin (3 μg/ml) (LKT Laboratories, St. Paul, MN) for the antibiotic selection of HeLa cells stably expressing CD63-GFP (CD63-GFP-HeLa).

### Isolation of exosomes

CD63-GFP-HeLa cells (3 × 10^6^ cells) were seeded on 100 mm dishes in α-MEM containing 10% exosome-free FBS (EXO-FBS, ATLAS biological, Fort Collins, CO, USA) for 3 days. The cell culture medium was collected, and secreted exosomes were isolated by Total Exosome Isolation (from cell culture media) (Invitrogen, Austin, TX, USA). The concentrations of isolated exosomes were described in terms of their protein concentrations, which were determined using a Pierce BCA protein assay kit (Thermo Fisher Scientific Inc., Rockford, IL, USA).

Isolation of exosomes was conducted using ultracentrifugation^39^. The collected cell culture medium was centrifuged (300 × *g*) for 10 min at 4 °C. The supernatant was centrifuged (2,000 × *g*) for 10 min at 4 °C and again centrifuged (10,000 × *g*) for 30 min at 4 °C to remove cell debris. The supernatant was then centrifuged (100,000 × *g*) for 30 min at 4 °C using an ultracentrifuge (Himac CP65β, Hitachi, Tokyo, Japan) in duplicate, and the pellet was collected in PBS.

### Western blot analysis

Isolated exosomes were added to lysis buffer (62.5 mM Tris-HCl (pH = 6.8), 2% SDS, 10% glycerol, 0.002% bromlopheno blue, 5% 2-mercaptoethanol). The boiled samples were separated by 10% SDS-PAGE, transferred to polyvinylidene fluoride (PVDF) membranes (GE Healthcare, Pittsburgh, PA, USA), and treated with anti-CD63 antibody (TS63, Abcam, Cambridge, UK), anti-GFP antibody (ab290, Abcam) and anti-CD9 antibody (EPR2949, Abcam). A secondary antibody labelled with horseradish peroxidase (anti-rabbit IgG HRP-linked whole antibody donkey, GE Healthcare) was used, and immunoreactive species were detected by an Enhanced Chemiluminescence (ECL) Plus Western Blotting Detection System (GE Healthcare). Immunoreactive species were detected at a position of approximately 50 kDa in SDS-PAGE analysis using anti-CD63 and anti-GFP antibodies.

### Complex of exosomes with cationic lipids and GALA peptide

Lipofectamine LTX diluted with α-MEM (total 20 μl) was added to a solution of exosomes and the GALA peptide in α-MEM (total 20 μl) and incubated for 20 min at room temperature. The mixture was then added to heat-inactivated FBS (20 μl) and serum-free α-MEM (140 μl), prior to cellular uptake assays.

### Confocal microscopy

Cells (2 × 10^5^ cells, 2 ml) were plated on a 35 mm glass dish (Iwaki, Tokyo, Japan) and incubated in cell culture medium (HeLa cells, α-MEM; CHO-K1 cells, Ham’s F-12) containing 10% FBS for 24 h at 37 °C under 5% CO_2_. After complete adhesion, the cells were washed with cell culture medium containing 10% FBS and treated with exosomal samples (200 μl/well). The cells were then washed with fresh cell culture medium and analysed using a FV1200 confocal laser scanning microscope (Olympus, Tokyo, Japan) equipped with a ×40 objective. To stain endosomes, HeLa cells were pre-stained with DiD (Invitrogen, 2.5% (v/v)) in 10% FBS containing α-MEM (200 μl/well) at 4 °C for 5 min. The cells were then washed with fresh cell culture medium, and treated with CD63-GFP-exosomes (20 μg/ml) with the combined treatment with Lipofectamine LTX (2% (v/v)) at 37 °C for 6 h, prior to observation by confocal microscope. To prevent endosomal pH-reduction, NH_4_Cl (50 mM) was added in exosomal samples as previously reported.^20^

### Flow cytometer

Cells (4.7 × 10^4^ cells, 1 ml) were plated on a 24 well microplate (Iwaki) and incubated in cell culture medium (HeLa cells, α-MEM; CHO-K1 cells, Ham’s F-12) containing 10% FBS for 24 h at 37 °C under 5% CO_2_. After complete adhesion, the cells were washed with cell culture medium containing 10% FBS and treated with exosomal samples (200 μl/well), prior to washing with phosphate buffered saline (PBS) (triple washing, 200 μl). The cells were then treated with 0.01% trypsin at 37 °C for 10 min, prior to the addition of PBS (200 μl), and centrifuged at 3,000 rpm (800 × *g*) for 3 min at 4 °C. After removal of the supernatant, the cells were washed with PBS (400 μl) and centrifuged at 3,000 rpm for 3 min at 4 °C. This washing cycle was repeated, and the cells were suspended in PBS (400 μl) and subjected to fluorescence analysis on a guava easyCyte (Merck Millipore, Billerica, MA, USA) flow cytometer using 488 nm laser excitation and a 525 nm emission filter. Living cells (10,000 cells/sample) for the detection of cellular fluorescence intensity were counted based on forward scatter and side scatter analyses.

### Electron microscope and zeta-potential

Suspended exosomes in PBS (30 μg/ml) were dropped on a carbon-coated grid (400 mesh) and washed with distilled water. Uranil acetate was applied to the grid and left for 10 sec at room temperature. Then, the reagent was removed with filter paper and dried, prior to imaging with a transmission electron microscope (TEM) (JEM1200EX, JEOL, Tokyo, Japan). The zeta-potential of the exosomes diluted in PBS (25 μg/ml) was determined using a zeta-potential analyser ELSZ-DN2 (Otsuka electronics, Osaka, Japan) according the manufacturer’s instructions.

### Preparation of FITC-labelled saporin

Two hundred microgram of saporin (saporin from *Saponaria officinalis* seeds, Sigma-Aldrich) dissolved in H_2_O (100 μl) was reacted with 2 equivalents of FITC (Sigma-Aldrich) dissolved in dimethyl sulfoxide (10 μl) and *N,N*-diisopropylethylamine (0.5 μl) at 30 °C for 2 h. For the removal of unreacted FITC, gel filtration on a Sephadex G-25 column (PD-10, GE Healthcare) was conducted prior to lyophilisation. The protein concentration was determined using a Pierce BCA protein assay kit (Thermo Fisher Scientific Inc.).

### Encapsulation of fluorescently-labelled dextran and saporin into exosomes

To load fluorescently-labelled dextran in exosomes, exosomes (25 μg) were mixed with Texas red-labelled dextran (Molecular Probes, Eugene, OR, USA), FITC-labelled dextran (70 kDa) (Sigma-Aldrich), or saporin (50 μg) in PBS (100 μl). After electroporation (poring pulse: twice pulse (5 msec), transfer pulse: five pulse (20 V, 50 msec)) in a 1 cm electroporation cuvette at room temperature using a super electropolater NEPA21 TypeII (NEPA genes, Tokyo, Japan), removal of unencapsulated Texas red-dextran, FITC-dextran, or saporin was accomplished by washing and filtration using Amicon Ultra centrifugal filters (100K device, Merck Millipore). Texas red-dextran and FITC-dextran loaded in exosomes were confirmed using a spectrofluometer (FP-6200, JASCO, Tokyo, Japan). The electroporation method was optimized for poring pulse (100, 200, 300 V), resulting in encapsulation of Texas red-dextran (80, 150, 110 ng/ml) in 20 μg/ml of exosomes, respectively. Therefore, we used an experimental poring pulse of 200 V in the electroporation steps. The efficiency of dextran encapsulation into exosomes was calculated to be 0.4%. The concentration of saporin encapsulated in 20 μg/ml exosomes was estimated to be around 180 ng/ml using the FITC-labelled saporin. The efficiency of saporin encapsulation into exosomes was calculated to be 0.5%.

### Cell viability (WST-1 assay)

Cell viability was analysed by a WST-1 (4-[3-(4-iodophenyl)-2-(4-nitrophenyl)-2H-5-tetrazolio]-1,3-benzene disulfonate) assay as previously described^40^. Cells (1 × 10^4^ cells, 100 μl) were incubated in 96 well microplates in cell culture medium (HeLa cells, α-MEM; CHO-K1 cells, Ham’s F-12) containing 10% FBS for 24 h at 37 °C under 5% CO_2_. The cells were then treated with exosomal samples (50 μl) for 24 h at 37 °C under 5% CO_2_. After the sample treatment, WST-1 reagents (10 μl) were added to each well, and the samples were incubated for 45 min at 37 °C. Absorbencies at 450 nm (A_450_) and 620 nm (A_620_) were measured, and the value obtained by subtracting A_620_ from A_450_ corresponded to the viable cell number.

## Additional Information

**How to cite this article**: Nakase, I. and Futaki, S. Combined treatment with a pH-sensitive fusogenic peptide and cationic lipids achieves enhanced cytosolic delivery of exosomes. *Sci. Rep.* **5**, 10112; doi: 10.1038/srep10112 (2015).

## Supplementary Material

Suplementary Information

## References

[b1] Tan A., Rajadas J. & Seifalian A. M. Exosomes as nano-theranostic delivery platforms for gene therapy. Adv. Drug Deliv. Rev. 65, 357–367 (2013). 22820532 10.1016/j.addr.2012.06.014

[b2] Vlassov A.V., Magdaleno S., Setterquist R. & Conrad R. Exosomes: current knowledge of their composition, biological functions, and diagnostic and therapeutic potentials. Biochim. Biophys. Acta. 1820, 940–948 (2012). 22503788 10.1016/j.bbagen.2012.03.017

[b3] van Dommelen S.M. *et al.* Microvesicles and exosomes: opportunities for cell-derived membrane vesicles in drug delivery. J. Control Release 161, 635–644 (2012). 22138068 10.1016/j.jconrel.2011.11.021

[b4] Conde-Vancells J. *et al.* Characterization and comprehensive proteome profiling of exosomes secreted by hepatocytes. J. Proteome Res. 7, 5157–5166 (2008). 19367702 10.1021/pr8004887 PMC2696236

[b5] Subra C. *et al.* Exosomes account for vesicle-mediated transcellular transport of activatable phospholipases and prostaglandins. J. Lipid Res. 51, 2105–2120 (2010). 20424270 10.1194/jlr.M003657 PMC2903822

[b6] Morelli A. E. *et al.* Endocytosis, intracellular sorting, and processing of exosomes by dendritic cells. Blood 104, 3257–3266 (2004). 15284116 10.1182/blood-2004-03-0824

[b7] Théry C., Ostrowski M., & Segura E. Membrane vesicles as conveyors of immune responses. Nat. Rev. Immunol. 9, 581–593 (2009). 19498381 10.1038/nri2567

[b8] Tian T. *et al.* Visualizing of the cellular uptake and intracellular trafficking of exosomes by live-cell microscopy. J. Cell. Biochem. 111, 488–496 (2010). 20533300 10.1002/jcb.22733

[b9] Svensson K.J. *et al.* Exosome uptake depends on ERK1/2-heat shock protein 27 signaling and lipid Raft-mediated endocytosis negatively regulated by caveolin-1. J. Biol. Chem. 288, 17713–17724 (2013). 23653359 10.1074/jbc.M112.445403 PMC3682571

[b10] Hannafon B.N., Ding W.Q. Intercellular communication by exosome-derived microRNAs in cancer. Int. J. Mol. Sci. 14, 14240–14269 (2013). 23839094 10.3390/ijms140714240 PMC3742242

[b11] Properzi F., Logozzi M., & Fais S. Exosomes: the future of biomarkers in medicine. Biomark. Med. 7, 769–778 (2013). 24044569 10.2217/bmm.13.63

[b12] Ogata-Kawata H. *et al.* H.Circulating exosomal microRNAs as biomarkers of colon cancer. PLoS One 9, e92921 (2014). 24705249 10.1371/journal.pone.0092921 PMC3976275

[b13] Wahlgren J. *et al.* Plasma exosomes can deliver exogenous short interfering RNA to monocytes and lymphocytes. Nucleic Acids Res. 40, e130 (2012). 22618874 10.1093/nar/gks463 PMC3458529

[b14] Kosaka N. *et al.* Exosomal tumour-suppressive microRNAs as novel cancer therapy: “exocure” is another choice for cancer treatment. Adv. Drug Deliv. Rev. 65, 376–382 (2013). 22841506 10.1016/j.addr.2012.07.011

[b15] Brambilla D., Luciani P., & Leroux J.C. Breakthrough discoveries in drug delivery technologies: the next 30 years. J. Control. Release 190, 9–14 (2014). 24794899 10.1016/j.jconrel.2014.03.056

[b16] Alvarez-Erviti L. *et al.* Delivery of siRNA to the mouse brain by systemic injection of targeted exosomes. Nat. Biotechnol. 29, 341–345 (2011). 21423189 10.1038/nbt.1807

[b17] Lai R. C., Yeo R. W., Tan K. H. & Lim S. K. Exosomes for drug delivery - a novel application for the mesenchymal stem cell. Biotechnol. Adv. 31, 543–551 (2013). 22959595 10.1016/j.biotechadv.2012.08.008

[b18] Tian T. *et al.* Dynamics of exosome internalization and trafficking. J. Cell. Physiol. 228, 1487–1495 (2013). 23254476 10.1002/jcp.24304

[b19] Tian Y. *et al.* A doxorubicin delivery platform using engineered natural membrane vesicle exosomes for targeted tumour therapy. Biomaterials 35, 2383–2390 (2014). 24345736 10.1016/j.biomaterials.2013.11.083

[b20] Kobayashi S. *et al.* Cytosolic targeting of macromolecules using a pH-dependent fusogenic peptide in combination with cationic liposomes. Bioconjug. Chem. 20, 953–959 (2009). 19388672 10.1021/bc800530v

[b21] Nakase I., Kogure K., Harashima H., & Futaki S. Application of a fusiogenic peptide GALA for intracellular delivery. Methods Mol. Biol. 683, 525–533 (2011). 21053154 10.1007/978-1-60761-919-2_37

[b22] Sokolova V. *et al.* Characterisation of exosomes derived from human cells by nanoparticle tracking analysis and scanning electron microscopy. Colloids Surf. B Biointerfaces 87, 146–150 (2011). 21640565 10.1016/j.colsurfb.2011.05.013

[b23] Hood J.L., San R.S., & Wickline S.A. Exosomes released by melanoma cells prepare sentinel lymph nodes for tumour metastasis. Cancer Res. 71, 3792–3801 (2011). 21478294 10.1158/0008-5472.CAN-10-4455

[b24] Takahashi Y. *et al.* Visualization and *in vivo* tracking of the exosomes of murine melanoma B16-BL6 cells in mice after intravenous injection. J. Biotechnol. 165, 77–84 (2013). 23562828 10.1016/j.jbiotec.2013.03.013

[b25] Hunt M. A., Currie M. J., Robinson B. A., & Dachs G. U. Optimizing transfection of primary human umbilical vein endothelial cells using commercially available chemical transfection reagents. J. Biomol. Tech. 21, 66–72 (2010). 20592869 PMC2884313

[b26] Subbarao N. K. *et al.* pH-dependent bilayer destabilization by an amphipathic peptide. Biochemistry 26, 2964–2972 (1987). 2886149 10.1021/bi00385a002

[b27] Li W., Nicol F., & Szoka F. C. Jr. GALA: a designed synthetic pH-responsive amphipathic peptide with applications in drug and gene delivery. Adv. Drug Deliv. Rev. 56, 967–985 (2004). 15066755 10.1016/j.addr.2003.10.041

[b28] Bostad M. *et al.* Light-triggered, efficient cytosolic release of IM7-saporin targeting the putative cancer stem cell marker CD44 by photochemical internalization. Mol. Pharm. 11, 2764–1776 (2014). 24960585 10.1021/mp500129t

[b29] Parolini I. *et al.* Microenvironmental pH is a key factor for exosome traffic in tumour cells. J. Biol. Chem. 284, 34211–34222 (2009). 19801663 10.1074/jbc.M109.041152 PMC2797191

[b30] Nakase I., Kobayashi S., & Futaki S. Endosome-disruptive peptides for improving cytosolic delivery of bioactive macromolecules. Biopolymers 94, 763–770 (2010). 20564044 10.1002/bip.21487

[b31] Simões S. *et al.* Gene delivery by negatively charged ternary complexes of DNA, cationic liposomes and transferrin or fusigenic peptides. Gene Ther. 5, 955–964 (1998). 9813667 10.1038/sj.gt.3300674

[b32] Simões S. *et al.* Mechanisms of gene transfer mediated by lipoplexes associated with targeting ligands or pH-sensitive peptides. Gene Ther. 6, 1798–1807 (1999). 10602375 10.1038/sj.gt.3301015

[b33] Simões S. *et al.* Transfection of human macrophages by lipoplexes via the combined use of transferrin and pH-sensitive peptides. J. Leukoc. Biol. 65, 270–179 (1999). 10088611 10.1002/jlb.65.2.270

[b34] Simões S. *et al.* Human serum albumin enhances DNA transfection by lipoplexes and confers resistance to inhibition by serum. Biochim Biophys Acta. 1463, 459–469 (2000). 10675522 10.1016/s0005-2736(99)00238-2

[b35] Futaki S. *et al.* Unique features of a pH-sensitive fusogenic peptide that improves the transfection efficiency of cationic liposomes. J. Gene Med. 7, 1450–1458 (2005). 16025556 10.1002/jgm.796

[b36] Nicol F., Nir S., Szoka F. C. Jr. Effect of cholesterol and charge on pore formation in bilayer vesicles by a pH-sensitive peptide. Biophys. J. 71, 3288–3301 (1996). 8968598 10.1016/S0006-3495(96)79521-8 PMC1233816

[b37] Nicol F., Nir S., Szoka F. C. Jr. Effect of phospholipid composition on an amphipathic peptide-mediated pore formation in bilayer vesicles. Biophys. J. 78, 818–829 (2000). 10653794 10.1016/S0006-3495(00)76639-2 PMC1300684

[b38] Choi H. S., Huh J., Jo W. H. pH-induced helix-coil transition of amphipathic polypeptide and its association with the lipid bilayer: electrostatic energy calculation. Biomacromolecules 7, 403–406 (2006). 16398543 10.1021/bm050579z

[b39] Théry C., Amigorena S., Raposo G. & Clayton A. Isolation and characterization of exosomes from cell culture supernatants and biological fluids. Curr. Protoc. Cell Biol. 30, Chapter 3, Unit 3.22 (2006). 10.1002/0471143030.cb0322s30 18228490

[b40] Nakase I. *et al.* Transformation of an antimicrobial peptide into a plasma membrane-permeable, mitochondria-targeted peptide via the substitution of lysine with arginine. Chem. Commun. 48, 11097–11099 (2012). 10.1039/c2cc35872g 23038156

