# Supplementary material for: Combined treatment with a pH-sensitive fusogenic peptide and cationic lipids achieves enhanced cytosolic delivery of exosomes
Source: Sci Rep. 2015 May 26;5:10112. doi: 10.1038/srep10112 (PMC4443764; doi:10.1038/srep10112)
Supplement: Suplementary Information [file srep10112-s1.pdf]

Scientific Reports

Supplementary information

Title:

Combined treatment with a pH-sensitive fusogenic peptide and cationic lipids achieves enhanced cytosolic delivery of exosomes

Authors:

Ikuhiko Nakase[1,*], Shiroh Futaki[2]

Affiliations:

[1]Nanoscience and Nanotechnology Research Center, Research Organization for the 21st Century, Osaka Prefecture University, Naka-ku, Sakai, Osaka 599-8570, Japan

[2]Institute for Chemical Research, Kyoto University, Uji, Kyoto 611-0011, Japan

*Correspondence and requests for materials should be addressed to I.N. (email: i-nakase@21c.osakafu-u.ac.jp).

Figure caption

Supplementary Figure 1. **Secretion of CD63-GFP-exosomes from HeLa cells.** (**a**) Confocal microscopic observation of CD63-GFP-HeLa cells or wildtype HeLa cells (without expression of CD63-GFP). Scale bar: 20 µm. (**b–d**) Western blot showing exosomes secreted from HeLa cells. The CD63 (**b**), GFP (**c**) and CD9 (**d**) exosome marker proteins were detected as described in the Materials section. Detection of immunoreactive species using anti-CD63 and anti-GFP were the same position at approximately 50 kDa. (**e**) TEM observation of isolated CD63-GFP-exosomes. Scale bar: 100 nm.

Supplementary Figure 2. **Cationic lipids increase the internalisation of exosomes into HeLa cells.** (**a**) Confocal microscopic observation of HeLa cells treated with CD63-GFP-exosomes (20 µg/ml) in the presence or absence of Lipofectamine LTX (1–4% (v/v)) for 24 h at 37 °C (blue: Hoechst33342, green: CD63-GFP-exosomes). Scale bar: 20 µm. (**b**) Colocalization of CD63-GFP-exosomes (20 µg/ml) in the presence of Lipofectamine LTX (2% (v/v)) and endosome marker DiD was analysed using a confocal microscope after the treatment for 6 h at 37 °C (green: CD63-GFP-exosomes, red: DiD). Arrows show representative colocalization of CD63-GFP-exosomes and DiD. Scale bar: 5 µm.

Supplementary Figure 3. **Enhanced cellular uptake of exosomes by treatment with cationic lipids.** (**a**) Confocal microscopic observation of CHO-K1 cells treated with CD63-GFP-exosomes (20 µg/ml) in the presence or absence of Lipofectamine LTX (2 or 4% (v/v)) for 24 h at 37 °C (blue: Hoechst33342, green: CD63-GFP-exosomes). Scale bar: 20 µm. (**b**) Relative cellular uptake of CD63-GFP-exosomes (20 µg/ml) in CHO-K1 cells in the presence or absence of Lipofectamine LTX (2 or 4% (v/v)) analysed using a flow cytometer under the same experimental conditions as (**a**). (**c**) Cytotoxicity in the combined treatment of exosomes (20 µg/ml) and Lipofectamine LTX (2–8% (v/v)) for 24 h at 37 °C on CHO-K1 cells analysed by a WST-1 assay. The data represent the averages (± SD) of three (**b**) and four (**c**) experiments.

Supplementary Figure 4. **Combination treatment with cationic lipids increases cellular uptake of exosomes.** (**a**) Relative cellular uptake of CD63-GFP-exosomes (1–20 µg/ml) in HeLa cells in the presence or absence of Lipofectamine LTX (0.5–2% (v/v)) for 24 h at 37 °C

analysed using a flow cytometer. (**b**) Cytotoxicity in the combined treatment of exosomes (1–10 μg/ml) and Lipofectamine LTX (0.5–4% (v/v)) for 24 h at 37 °C on HeLa cells analysed by a WST-1 assay. The data represent the averages (± SD) of three (**a**) and four (**b**) experiments.

Supplementary Figure 5. **Concentration of GALA peptide affects the cytosolic diffusion efficiency.** (**a**) Confocal microscopic observation of HeLa cells treated with exosomes (without expression of CD63-GFP, 5 μg/ml) and FITC-GALA (0.05, 0.5 or 2.5 μM) in the presence of Lipofectamine LTX (0.5% (v/v)) for 6 h at 37 °C (blue: Hoechst33342, green: FITC-GALA). Scale bar: 20 μm. (**b**) Observation of HeLa cells treated with CD63-GFP-exosomes (5 μg/ml) and GALA (without fluorescent label, 0.5 μM) in the presence of Lipofectamine LTX (0.5% (v/v)) for 6 h at 37 °C (green: CD63-GFP-exosomes). Scale bar: 10 μm. (**c**) HeLa cells were treated with exosomes (without expression of CD63-GFP, 5 μg/ml), FITC-GALA (0.5 μM), and Lipofectamine LTX (0.5% (v/v)) in the presence or absence of NH_4Cl (50 mM) for 6 h at 37 °C, prior to confocal microscope observation. Scale bar: 20 μm. (**d**) Confocal microscopic observation of HeLa cells treated in combination with TR-dex encapsulated exosomes (5 μg/ml), FITC-GALA (0.5 μM), and Lipofectamine LTX (0.5% (v/v)) in the presence or absence of NH_4Cl (50 mM) for 6 h at 37 °C (green: FITC-GALA, red: TR-dex). Scale bar: 10 μm.

Supplementary Figure 6. **Cellular uptake of encapsulated Texas red-dextran in exosomes treated with cationic lipids and GALA peptide.** Confocal microscopic observation of CHO-K1 cells treated with a combination of TR-dex encapsulated exosomes (20 μg/ml) and FITC-GALA (0, 2 or 10 μM) in the presence of Lipofectamine LTX (2% (v/v)) for 6 h at 37 °C (green: FITC-GALA, red: TR-dex). Scale bar: 20 μm.

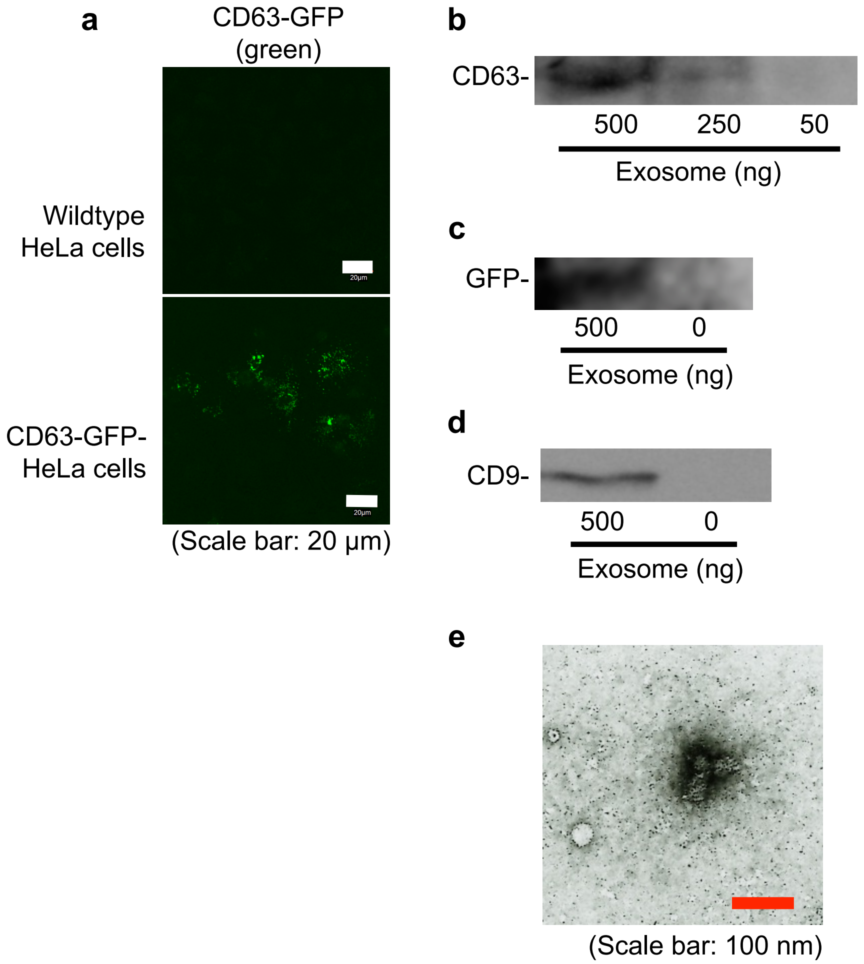

Supplementary Figure 1

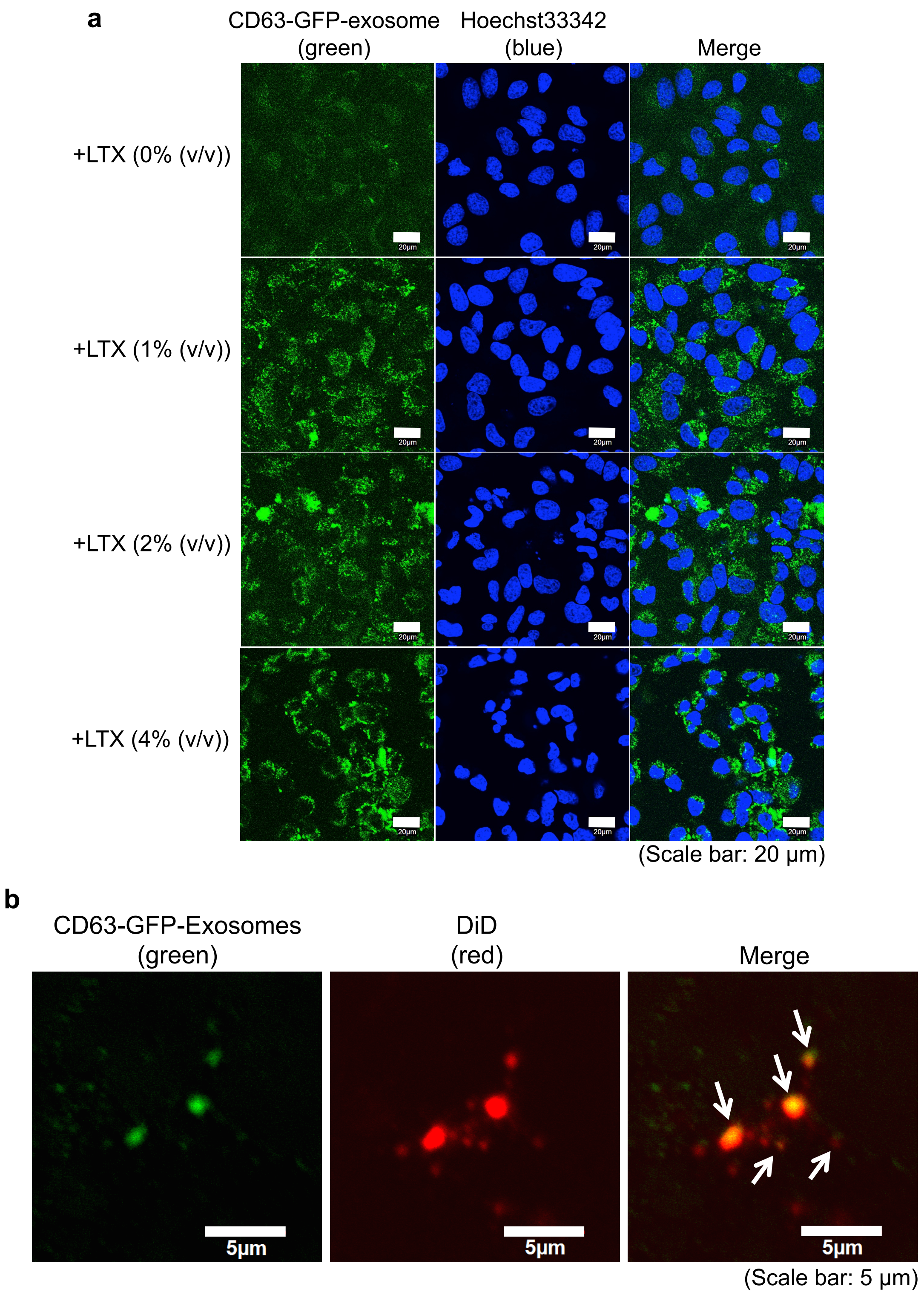

Supplementary Figure 2

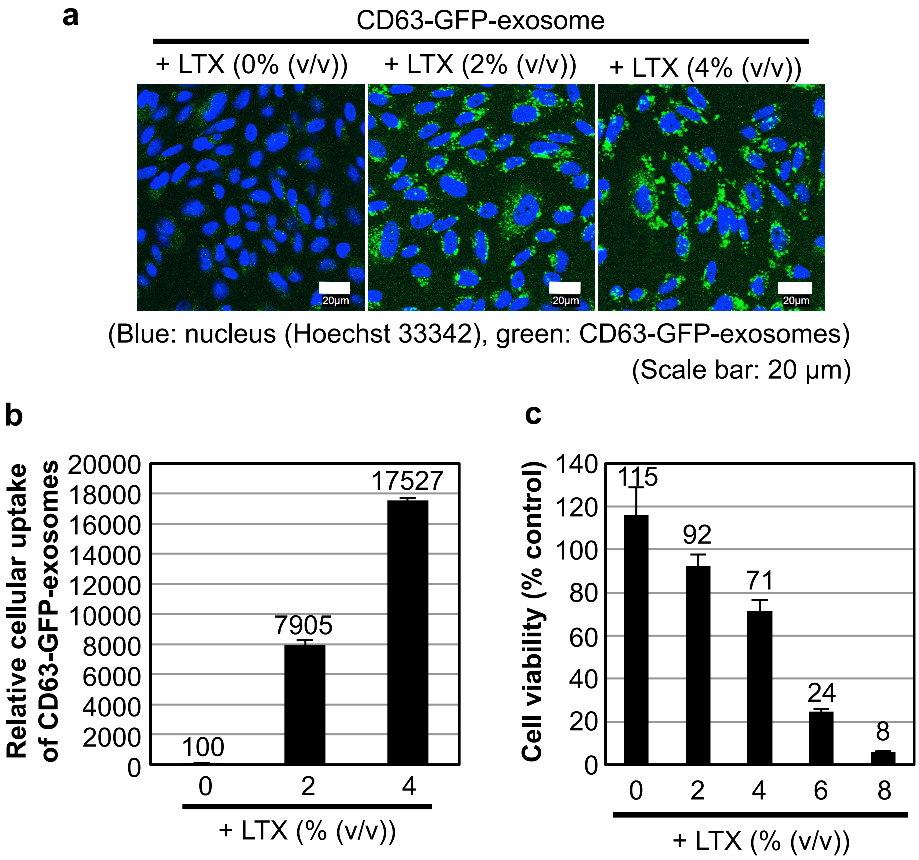

Supplementary Figure 3

a

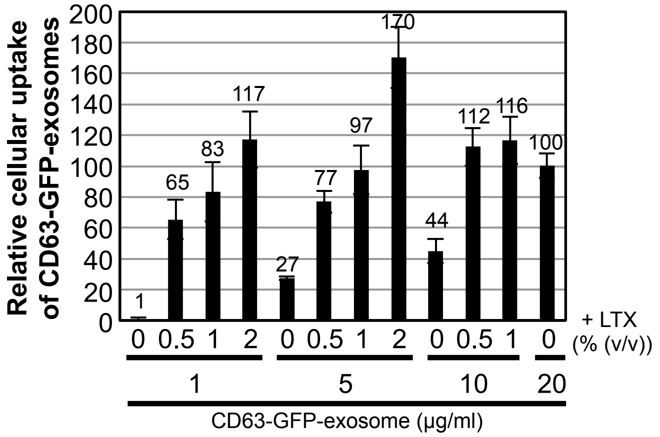

b

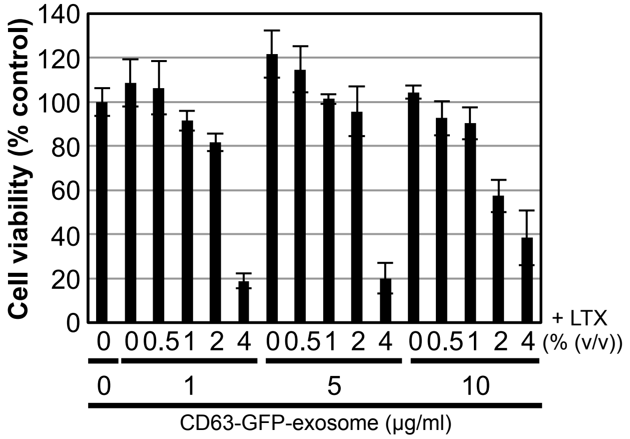

Supplementary Figure 4

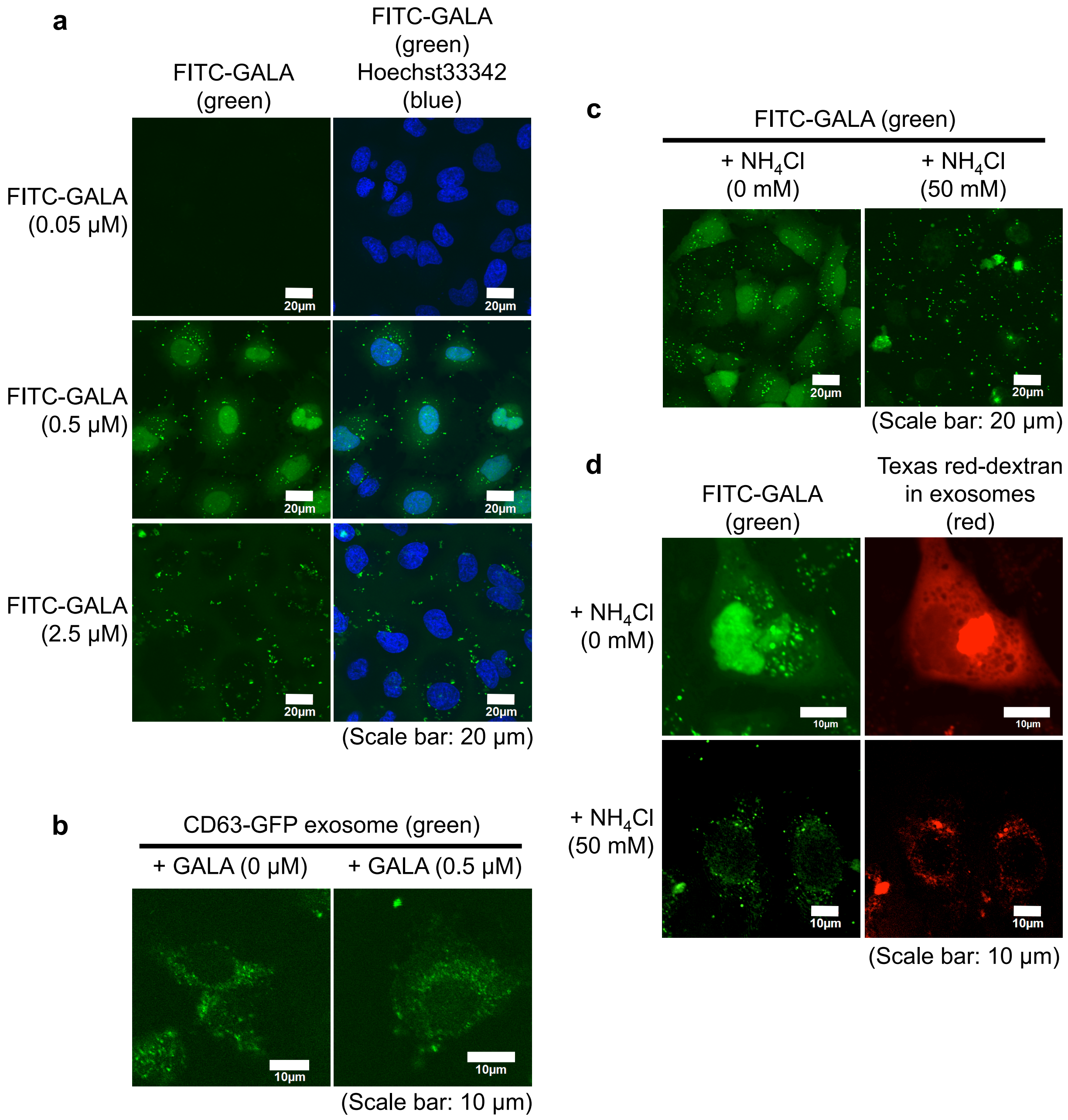

Supplementary Figure 5

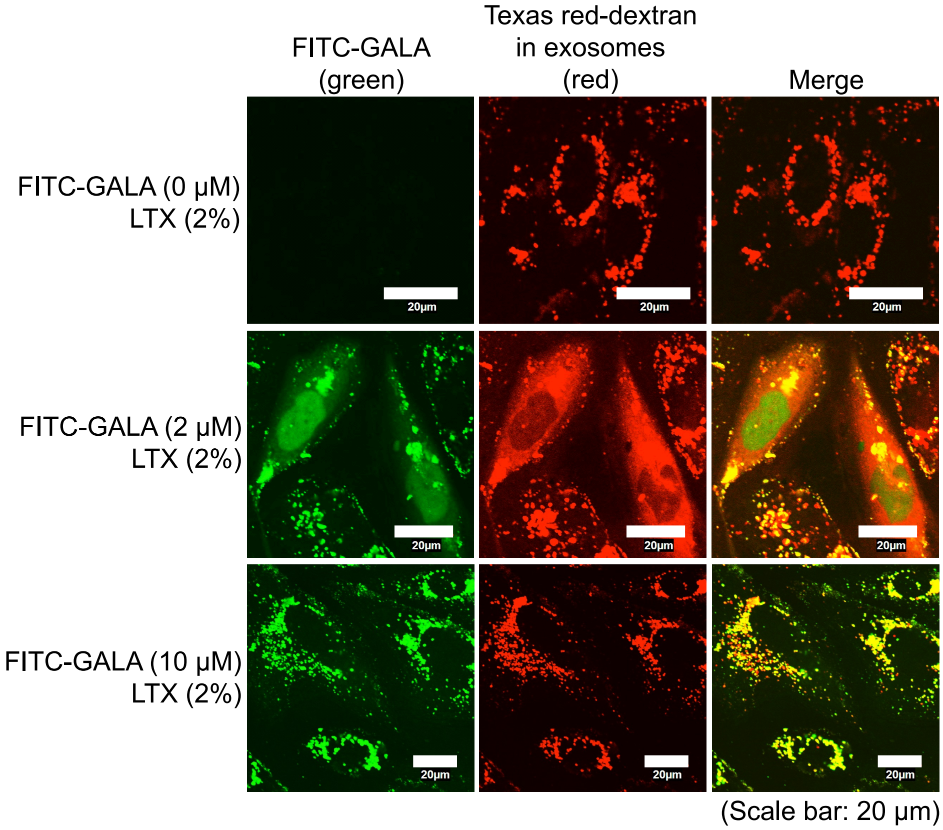

Supplementary Figure 6
